# Universal health coverage and community engagement

**DOI:** 10.2471/BLT.17.202382

**Published:** 2018-07-16

**Authors:** Asiya Odugleh-Kolev, John Parrish-Sprowl

**Affiliations:** a World Health Organization, avenue Appia 20, 1211 Geneva 27, Switzerland.; b Global Health Communication Center, Indiana University, Indianapolis, United States of America.

Achieving universal health coverage (UHC) and the sustainable development goals (SDGs) requires health systems to shift from an almost exclusively vertical, top-down and curative paradigm to one that places people at the centre of health services.

Here we reflect on how efforts towards UHC could offer an opportunity to address those aspects within health systems that continue to hinder efforts to meaningfully engage with patients, their families and local communities. The backbone of these efforts should be a health workforce that is skilled in engagement, responsive to local context and to the needs and expectations of those using their services. 

The complexity of health issues in today’s globalized world challenges the fragmented and often isolated practices in both contemporary medicine and public health in much of the world.^1^ For example, managing chronic diseases and multimorbidities and responding to outbreaks of infectious diseases challenges health systems, as they require collaborative action at different levels of the system and across multiple stakeholders, sectors and countries. Diverse disciplines, professions and institutions should work together to find more sustainable and locally owned solutions and practices to address fundamental issues, such as equity and the social determinants of health.^2^ With UHC as a major goal for health reform in many countries and a priority for the World Health Organization (WHO), there is an opportunity to focus on how such high-level consensus can be translated into meaningful action.^3^ Therefore, it is timely to consider the emerging role of non-traditional areas for health systems, such as community engagement, within UHC, in particular how community engagement can shape the delivery of quality health services that are safe, effective, efficient, equitable, people centred and that integrate care.^4^

## UHC and community engagement

The health sector has well-documented experiences in community participation and engagement, not all of them successful.^5^^,^^6^ In the declaration of Alma-Ata in 1978, Member States agreed that community participation was a fundamental component of primary health care. Since then, health researchers, practitioners and policy-makers have worked to develop a meaningful set of practices that contribute to strengthening community participation. The term community engagement, as opposed to participation, emerged from the field of health research and focused on the deliberate integration of communities into the design and implementation of research activities. Researchers, being both reflective and reflexive agents in this process, meaning, aware that their presence can alter circumstances, were fundamental to this approach.^7^ Community engagement was introduced in the 2013–2016 Ebola virus disease outbreak in recognition of the important role of response staff and their ability to engage with communities, in contrast to social mobilization or behaviour-change interventions. In today’s world, when health workers may not reside in the communities where they work, knowing how to work with communities in ways that build upon their culture, knowledge and experiences is important. In its most extreme, the manifestation of community fear and distrust has tragic consequences, such as the killings of health workers.^8^

Engagement and empowerment of health service users and community members also re-emerged as a core strategy in the WHO Framework on Integrated People-Centred Health Services,^9^ which was formally adopted by Member States in 2016. Yet, consensus is lacking on who the community is, what community engagement means, who is responsible for this engagement and how it is done and measured. To understand why there is such ambiguity, the underlying assumptions and paradigms that underpin mindsets and professional practice must be analyzed.^10^

## Creating a new paradigm

Traditional approaches to engaging communities through health providers have focused on better information provision and health messaging, and on the development of the providers’ communication skills. On the users’ side, this approach has prioritized increasing participation in decision-making and empowerment, either through helping patients, families and communities to develop enough literacy to use and navigate the health system or through mechanisms that promote social accountability and citizens’ rights. Neither side of this traditional approach reflect how health systems and communities relate to each other. To move towards a more meaningful understanding of what community engagement is and how it works, several changes need to take place.

First, we need to recognize that health systems have a fundamental responsibility and obligation for engaging with patients, their families, local communities, as well as a range of stakeholders, partners and sectors. Health systems should not solely rely on community health workers, despite their successful contribution to delivering vital services to the most vulnerable. Health systems already engage with communities at multiple levels, including: (i) settings, for instance in clinics, hospitals and health posts; (ii) public health functions and cadres, for example surveillance and community health workers; and (iii) consultative and accountability mechanisms, such as human rights, community representative bodies and councils. Relationships with communities develop and continue to be shaped within these contexts. When trust is broken, whether because of poor-quality services, lack of services, misuse of funds or discrimination, regaining this trust will take more than sending experts who might be unfamiliar with the locale and its culture, or cleverly packaged messages and materials. Trust will be re-established through the daily delivery of care and services. The fact that engagement is relational and bridges the supply and demand sides of health-care provision continues to be missed. As politicians and policy-makers commit to reorienting their health systems to be patient-centred, provide continuity of care and incorporate physical and mental health, they inadvertently reinforce the dichotomy between supply and demand. The focus on data and knowledge management by default prioritizes external levers for change, such as patient advocacy and civil society groups, rather than internal ones.^11^

Second, a growing body of scientific evidence suggests the need to develop greater focus on the dynamic interrelationship between brain and body functions, and how connections are made in interpersonal interaction. The relationships between health-care professionals and community members have an impact on the health and well-being of both groups. The outcomes of these interactions will also influence and determine how the health system performs. Consequently, community engagement will be more effective if it is based on an understanding of human engagement and development that recognizes the physiological, emotional, mental and social interconnection of people. We need to build on this research and generate evidence that applies new knowledge and understanding to further improve health coverage and efficacy.^12^

Third, the most important change that could facilitate integrating community engagement in UHC is proper funding for engagement interventions, supported by mechanisms of governance that allow for transparency, accountability and representation. Resources will need to be redistributed and channelled into community engagement as a cross-cutting technical area to support the generation of evidence for strong policy recommendations. Given the increasing demands being made on health systems around the world, investing in community engagement makes sense as it supports services and systems to maximise available resources. Donors and researchers should support the small investments needed to develop community engagement, which is critical to the future of public health and success of UHC.

## Integrating community engagement

No matter how much or how often communities are empowered, UHC will not be achieved until health systems and their diverse stakeholders are ready to engage with each other in effective ways. It is no longer about how much health professionals know, but about how they use their knowledge and work with and through others that makes a difference.^13^

Community engagement describes the human aspect of the health system; it calls for a combined set of interventions that include: (i) tackling health systems’ culture through establishing corporate values and investing in leaders and managers who collaborate, coach and create enabling work environments; (ii) reflective practices and tools that help staff self-regulate and manage daily interpersonal and group interactions; and (iii) integrating qualitative methods and tools from the social sciences into job functions to support contextualization and adaptation of health interventions and services.

Health systems and communities are in continuous and interdependent action. If community engagement becomes a focus for UHC efforts, it could finally push the health sector from an almost exclusively transactional model into one that recognizes that health and well-being are co-produced, and that empowers both health-care providers and communities. This means that governments, donors and researchers have to address institutional culture and invest in so-called soft skills, which a growing body of scientific research recognizes as vital for success of UHC.^14^ These skills ensure that health professionals can collaborate and safely connect to others with compassion and purpose. A workforce able to maintain receptive states, build relationships, manage group processes and synthesize quantitative and qualitative data can support health services to be integrated, coordinated, adaptive and responsive. These same skills and competencies connect and activate the collective intelligence that is distributed throughout the health system, which encourages innovation and fosters resilience in people and health systems.
